# Supplementary material for: Identification of Freezing-Responsive microRNAs and Their Targets in Chinese Jujube by Small RNA and Degradome Sequencing
Source: Int J Mol Sci. 2026 Sep 4;27(17):7912. doi: 10.3390/ijms27177912 (PMC13566890; doi:10.3390/ijms27177912)
Supplement: Supplementary file 1 [file ijms-27-07912-s001.zip › Supplementary Material S2/novel_miR_37.pdf]

Provisional ID	:	novel_miR_37
Score total	:	38.2
Score for star read(s)	:	3.9
Score for read counts	:	36.3
Score for mfe	:	0.2
Score for randfold	:	-2.2
Score for cons. seed	:	
Total read count	:	83
Mature read count	:	80
Loop read count	:	0
Star read count	:	3

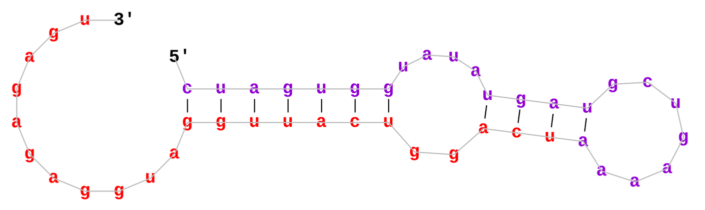

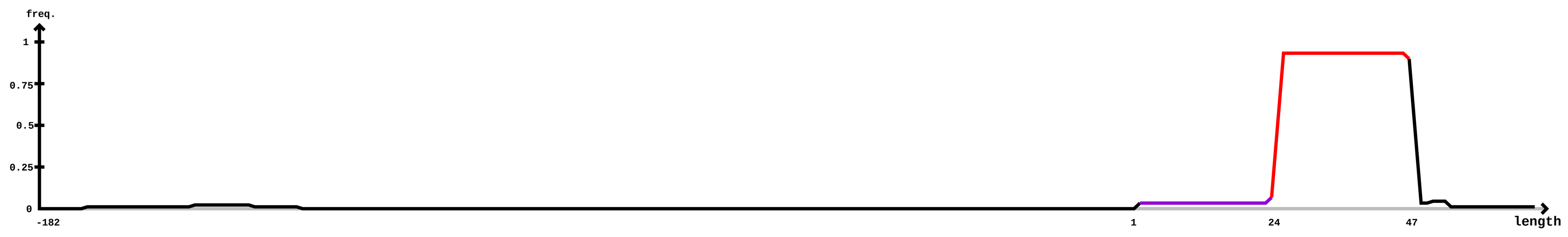

Star Mature

```
5'- accauguguuauaaaauuggaggcauuuugacguagaacuuucuggaacuugauauauagauugauuggggauaauuuuuaucaaaagaauggaguagaugaaauauuguuggugugggucuguauguuauuuauugacuucuuaauuguuuccaauugcagauauggugcuugcaaauaagcuagugguauaugaugcugaaaaucaggucauuggauggagagaguacaacuguaagcaucuuugauu -3' obs
    accauguguuauaaaauuggaggcauuuugacguagaacuuucuggaacuugauauauagauugauuggggauaauuuuuaucaaaagaauggaguagaugaaauauuguuggugugggucuguauguuauuuauugacuucuuaauuguuuccaauugcagauauggugcuugcaaauaagcuagugguauaugaugcugaaaaucaggucauuggauggagagaguacaacuguaagcaucuuugauu exp
    .((((((.(.....(((((((((((..(((....((((...((.......((((((((((((.((....((((((((((((.............))))))).))))))...)).)))))))))))))......)).))))))).))))))))))..).))))))(((((((.....(((..(.((..(((((.((((...)))))))))...)).)....))).....)))))))).........  reads  mm  sample
    ......uguuauaaaauuggaggcauuuugacgu..........  1  0  JCK-1
    ........................auuuugacguGgaacuuu..........  1  1  JT-2
    ..........cuagugguauaugaugcugaaaa..........  3  0  LT-2
    ..........auUaggucauuggauggagagag..........  3  1  LT-2
    ..........ucaggucauuggauggagagagu..........  40  0  LCK-2
    ..........ucaggucauuggauggagagagC..........  1  1  LCK-2
    ..........ucaggucauuggauggagagagu..........  36  0  LCK-1
    ..........caggucauuggauggagagaguacaacu..........  2  0  LCK-3
    ..........caggucauuggauggagagaguacaacu..........  1  0  JCK-2
    ..........Ccuguaagcaucuuugauu  1  1  LT-3
```
